# Shifts in reproductive assurance strategies and inbreeding costs associated with habitat fragmentation in Central American mahogany

**DOI:** 10.1111/j.1461-0248.2012.01752.x

**Published:** 2012-05

**Authors:** Martin F Breed, Michael G Gardner, Kym M Ottewell, Carlos M Navarro, Andrew J Lowe

**Affiliations:** 1 Australian Centre for Evolutionary Biology and Biodiversity (ACEBB) and School of Earth and Environmental Sciences, University of AdelaideNorth Terrace, Adelaide, South Australia 5005; 2 E-mail: martin.breed@adelaide.edu.au; 3 School of Biological Sciences, Flinders UniversityBedford Park, Adelaide, South Australia; 4 Department of Ecology and Evolutionary Biology, Tulane UniversitySaint Charles Avenue, New Orleans, Louisiana 70118; 5 Universidad Nacional, Instituto de Investigaciones y Servicios Forestales86-3000 Heredia, Costa Rica; 6 State Herbarium of South Australia, Science Resource Centre, Department of Environment and Natural ResourcesHackney Road, SA 5005, Australia

**Keywords:** Common garden experiment, global change, habitat fragmentation, inbreeding depression, logging, mating system

## Abstract

The influence of habitat fragmentation on mating patterns and progeny fitness in trees is critical for understanding the long-term impact of contemporary landscape change on the sustainability of biodiversity. We examined the relationship between mating patterns, using microsatellites, and fitness of progeny, in a common garden trial, for the insect-pollinated big-leaf mahogany, *Swietenia macrophylla* King, sourced from forests and isolated trees in 16 populations across Central America. As expected, isolated trees had disrupted mating patterns and reduced fitness. However, for dry provenances, fitness was negatively related to correlated paternity, while for mesic provenances, fitness was correlated positively with outcrossing rate and negatively with correlated paternity. Poorer performance of mesic provenances is likely because of reduced effective pollen donor density due to poorer environmental suitability and greater disturbance history. Our results demonstrate a differential shift in reproductive assurance and inbreeding costs in mahogany, driven by exploitation history and contemporary landscape context.

## Introduction

**Figure 1:**
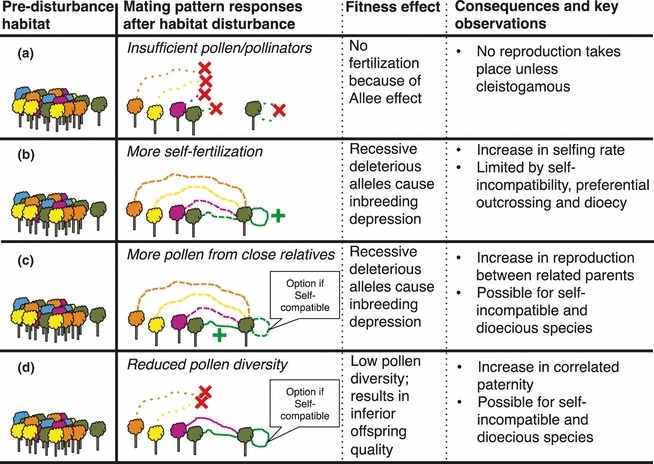
Possible mating system responses of trees to habitat disturbance, including (a) insufficient pollen/pollinators; (b) increased selfing; (c) increased biparental inbreeding; (d) decreased pollen diversity. Genetically similar trees are represented by colours. Pollination is indicated by lines that show failed pollination by coloured dotted lines followed by a red cross (×); reduced pollination by coloured dotted lines; normal pollination by solid coloured lines; a relative increase in pollination by coloured lines with a green plus (+). Responses (b, increased selfing), (c, increased biparental inbreeding) and (d, decreased pollen diversity) are not mutually exclusive for hermaphroditic and self-compatible species.

Forests form key global ecosystems that humans have utilised for millennia, but many have experienced unsustainable exploitation resulting in disrupted ecosystem processes (e.g. pollination; Fig. 1). Effects of human disturbance (e.g. clearing, selective logging) on tree mating patterns have been well studied (Eckert *et al.* 2010), including numerous neotropical examples (Lowe *et al.* 2005; Ward *et al.* 2005). Both self-compatible and self-incompatible species are expected to experience fitness declines through reduced mate availability with disturbance (Fig. 1, response a). Additionally, disturbances typically manifest as increased selfing for self-compatible species (Fig. 1, response b), and as increased reproduction between related parents for self-compatible and self-incompatible species (Fig. 1, response c; hereafter biparental inbreeding), both leading to fitness reductions due to inbreeding depression (Szulkin *et al.* 2010). Regardless of mating system, inbreeding depression is more commonly expressed in more stressful environments (Fox & Reed 2010) and is expected to become more severe as environment-dependent stress increases due to global change (Beaumont *et al.* 2011). For species within these altered habitats, new shifts in reproductive assurance and fitness costs associated with inbreeding may be observed (Herlihy & Eckert 2002; Kalisz *et al.* 2004).

In agricultural landscapes, the practice of retaining pasture trees produces a network of spatially isolated trees. Mating pattern disruption is expected to be most severe in these artificially low-density tree systems, particularly for animal-pollinated species (Ottewell *et al.* 2009; Breed *et al.* 2011). Indeed, several authors have reported dramatic fitness declines for trees in highly isolated contexts and have generally attributed these declines to inbreeding depression (Murawski & Hamrick 1991; Gribel *et al.* 1999). However, many studies investigating fitness impacts of tree isolation due to habitat disturbance lack sufficient sampling breadth to overcome potentially confounding population-specific effects. Additionally, these studies rarely consider mating system responses other than selfing, often neglecting correlated paternity and biparental inbreeding, thus limiting conclusions on the process driving changes in fitness.

Habitat disturbance is also expected to reduce the diversity of pollen received into tree canopies (Fig. 1, response d; Cascante *et al.* 2002; Fuchs *et al.* 2003). Consequently, benefits of pollen diversity (i.e. acquisition of ‘good genes’ associated with the augmentation of genetic diversity; Yasui 1998) are expected to decline in disturbed areas. The routinely estimated mating system parameter, correlated paternity (*r*_p_), indirectly measures pollen diversity and is inversely related to the effective number of pollen donors in sampled pollen clouds (Sork & Smouse 2006). Thus, correlated paternity is expected to increase in human-altered landscapes, as fewer pollen donors are present in the landscape and, as predicted by optimal foraging theory (Charnov 1976), animal pollinators stay longer in more isolated trees and move between neighbouring trees. However, surprisingly few studies have assessed the fitness consequences of correlated paternity changes resulting from habitat disturbance. Where correlated paternity and fitness have been assessed together (e.g. Rocha & Aguilar 2001; Cascante *et al.* 2002; Fuchs *et al.* 2003), fitness assessments have been undertaken over periods of time that were insufficient to rule out alternative explanations (e.g. maternal effects; Charlesworth 1988; Donohue 2009).

Despite some well-documented examples of changes in mating patterns resulting from tree isolation, there is still a scarcity of empirical data investigating the relative importance of individual mating patterns on fitness (i.e. selfing, biparental inbreeding, correlated paternity). The few studies that have conducted assessments of multiple mating pattern parameters together with fitness have assessed only a single population and have relied on comparisons of study group means (e.g. isolated vs. continuous forest contexts) rather than family means (i.e. means of offspring from a single mother tree). By deriving mating system parameters for families rather than groups of families, variance increases due to reduced sample sizes, but this approach can be used to derive statistical relationships between individual mating system parameters and fitness, rather than relying on *post hoc* comparisons of mating system values and fitness means. The larger number of comparisons within and between groups balances the increased variance observed for family, compared with population, level values. This approach can also be used to assess mating system changes in populations across the range of a species.

Due to its long history of timber exploitation in Central America, genetic consequences of habitat disturbance have been studied extensively for mahogany species in the genus *Swietenia*–a neotropical, small-insect pollinated (e.g. moths, bees), monoecious tree (Gillies *et al.* 1999; White *et al.* 2002; Lowe *et al.* 2003; Novick *et al.* 2003). Studies of spatial genetic structure of *Swietenia* suggest that pollen flow is limited because of small-insect pollination (Lowe *et al.* 2003). Consequently, *Swietenia* species may be susceptible to genetic drift following habitat disturbance. However, *Swietenia* species should be partially genetically buffered from habitat disturbance effects by strong outcrossing (Lemes *et al.* 2007), which may reduce a loss of genetic diversity by inbreeding avoidance. In addition, Navarro & Hernandez (2004) and Navarro *et al.* (2011) have demonstrated strong fitness impacts due to disturbance for *Swietenia macrophylla* King. These authors noted strong region-specific responses, best described when grouping trees into dry and mesic provenance types. On the basis of these observations, the authors speculate that the adaptive status of *S. macrophylla* was either the result of recent adaptive shifts or a shared evolutionary history under similar environmental conditions. However, without an analysis of genetic data, which can shed light on the mating system dynamics of populations, Navarro & Hernandez (2004) and Navarro *et al.* (2011) were not able to resolve the processes driving variation in fitness.

Here, we report an analysis of genetic variation in *S. macrophylla* progeny sourced from forest and isolated tree contexts in 16 Central America populations, spanning seven countries, that had previously been raised over a five-year period in a common garden experiment (reported by Navarro & Hernandez 2004 and Navarro *et al.* 2011). Rearing progeny to full maturity (> 20 years) would be ideal to best understand genetic and mating system impacts on fitness in this long-lived tree. However, 5 years is longer than is usually reported in these types of studies and should allow an examination of differences that probably affect growth in later life (beyond maternal effects). We predict that increased isolation will associate with increased selfing, increased biparental inbreeding (although no change or decreased biparental inbreeding is expected if isolation exceeds genetic neighbourhood size) and increased correlated paternity across the study populations (summarised in Fig. 1). We then predict that greater inbreeding and/or correlated paternity should negatively impact progeny growth (as an indicator of progeny fitness; Yasui 1998; Szulkin *et al.* 2010; Fig. 1). However, the region-specific growth responses demonstrated by Navarro & Hernandez (2004) and Navarro *et al.* (2011) suggest that factors governing progeny growth may vary across the study region. Thus, we predict that regional-level variation in environmental (e.g. latitude; Colautti *et al.* 2009), population (e.g. effective density) and/or exploitation history factors may drive variation in mating system parameters and growth.

We demonstrate that increased selfing and correlated paternity both negatively impact progeny growth, but that variation in these mating system parameters impacts growth differentially in rainfall-determined seed provenances (i.e. mesic or dry provenances). Using these data, we attempt to identify the mechanisms underlying progeny changes in growth to habitat disturbance, implementing a rigorous analytical framework that should overcome previous methodological shortfalls. Finally, we explore implications of habitat disturbance-driven shifts in inbreeding costs and reproductive assurance for tree species in a changing world.

## Materials and methods

### Collection methods

**Figure 2:**
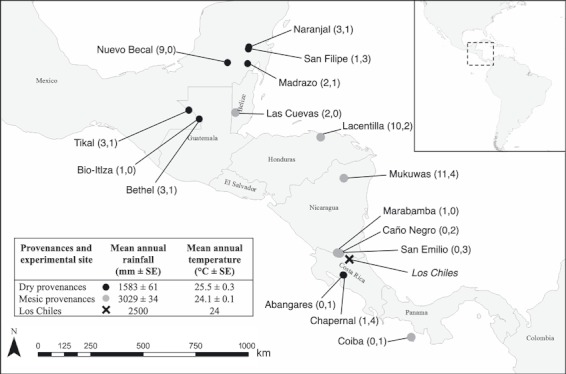
Study area map showing common garden experiment location (×), sample population locations from mesic (grey filled circles) and dry (black filled circles) provenances. Provenance-context sample sizes shown in parentheses (forest and isolated trees). Inset Table shows mean (mm ± standard error) annual rainfall of provenance-context groups. Inset map shows location of study region, highlighting study area (box).

Open-pollinated progeny used for field trials were collected from 16 natural populations across seven countries throughout the range of *S. macrophylla* in Central America (Fig. 2). In each population, mature trees (> 20 years of age) had seeds sampled according to methods described by Navarro *et al.* (2002), where trees were sampled along transects with a minimum distance of 100 m between trees. The number of trees sampled per population varied according to population size and accessibility. Approximately 20 fruits were collected per tree, each containing approximately 50 viable seeds. Approximately five seeds per fruit were germinated and reared for planting into the common garden experiment (details below). Mother trees were classified as being in an isolated context when no conspecifics were observed within 500 m, or as being from a forest context when conspecifics were present within 500 m and were located in either large remnant forests or forest patches (Navarro *et al.* 2002, 2011). To account for confounding population effects (e.g. adaptation), populations were grouped into high- or low-rainfall provenances following Navarro & Hernandez (2004) and Navarro *et al.* (2011). These provenances had comparable densities of trees estimated from data from Gillies *et al.* (1999; mean density: mesic provenances = 1.28 trees ha^−1^; dry provenances = 1.23 trees ha^−1^).

### Common garden experiment

To observe variation in progeny growth, approximately nine individuals from each of 71 mother trees were germinated and planted in a common garden experiment at Los Chiles, Alajuela, Costa Rica (Fig. 2) and measured over a five-year period. Los Chiles has tropical and mesic climate with approximately 2500 mm of mean annual rainfall and a mean annual temperature of 24 °C. A generalised randomised block design (Addelman 1969) was applied, with rows spaced every 3 m and seedlings spaced 3 m within rows. The height of the _i_th seedling from the _b_th block (*h*_ib_) and circumference at ground level (*c*_ib_ = 2π*r*_ib_) were estimated and used to estimate seedling conical volume, *â*_ib_ = 1/3π*r*_ib_^2^*h*_ib_. The difference between the mean volume of the _b_th block (*â*_b_) and the mean across all blocks (*ā*) was used to adjust *â*_ib_ to account for variation among blocks by _adjusted_*â*_ib_ = *â*_ib_ − (*â*_b_ − *ā*), and was averaged within families (_adjusted_*â*_ib_), hereafter ‘growth’. Using growth as a proxy for progeny fitness has limitations, as established seedlings may have experienced strong selection at pre- or early post-zygotic stages. In this study, germination rates were not observed to be different across progeny classes, but detailed data were not available for further analysis.

### Genetic analysis

DNA was extracted from leaves collected from seedlings using the Macherey-Nagel Nucleospin Plant II Kit at the Australian Genome Research Facility (AGRF, Adelaide, Australia). Each progeny was genotyped using seven dinucleotide microsatellite loci (SM01, SM22, SM31, SM32, SM40, SM46, SM51), described in Lemes *et al.* (2002). An adjusted PCR protocol (compared to Lemes *et al.* 2002) was used by amplifying each locus separately, using 0.25 μM final concentration of BSA and HotMaster™ Taq (Eppendorf, Hamburg, Germany). PCR products were pooled and LIZ500 size standard was added before separation on an AB3730 genetic analyser with a 36 cm capillary array (Applied Biosystems, Foster City, MA, USA). Alleles were sized using GeneMapper software (Applied Biosystems) and double-checked manually.

Null alleles result in false homozygotes and are expected to reduce estimates of observed heterozygosity, increase selfing, biparental inbreeding and correlated paternity. While null alleles have not previously been reported at these microsatellite loci (Lemes *et al.* 2002, 2007; Novick *et al.* 2003), we assessed the sensitivity of our results to the presence of null alleles. We estimated the frequency of null alleles at each locus in both wet and dry provenances using maternal genotypes in MICRO-CHECKER (Oosterhout *et al.* 2004). We detected significant levels of null alleles at two loci in both provenances (33% and 24% for SM46 and 10% and 14% for SM51 in mesic and dry provenances respectively). For loci with significant null alleles, we used MICRO-CHECKER to adjust datasets for the presence of these null alleles. We then compared F_IS_ estimates (disparity between expected and observed heterozygosity) for observed and null allele-adjusted datasets in both provenances for the loci that had significant null alleles in FSTAT (Goudet 1995). Additionally, we wished to determine the frequency of provenance-specific null alleles required to give comparable genetic results across provenances (i.e. to match mating system and observed heterozygosity in both dry and mesic provenances). To do this, we randomly inflated null allele frequencies for offspring data and re-ran mating system and heterozygosity analyses (see Appendix S1 for a full description of null allele methods and results).

### Mating system and genetic diversity estimation

The mating system parameters – multilocus outcrossing rate (*t*_m_), biparental inbreeding (*t*_*m*_ *− t*_*s*_) and correlated paternity (*r*_p_) – were estimated in MLTR (Ritland 2002). To calculate parameter variance for groups of families (i.e. provenances, isolation contexts, populations), families were bootstrapped 1000 times (families are groups of offspring from a known mother tree). To calculate parameter variance for families, individuals within families were bootstrapped 1000 times.

Observed family multilocus heterozygosity (*Ĥ*_j_) was estimated following Szulkin *et al.* (2010) and scaled to between 0 and 1 to account for missing data by *Ĥ*_j_ = Σ*H*_ij_/*n*_j_, where *H*_ij_ is progeny multilocus heterozygosity for the _i_th individual in the _j_th family, *n*_j_ is the number of progeny in the _j_th family, and *H*_ij_ = Σ*h*_ik_/*n*_k_ where *h* is a heterozygote for the _i_th individual at successfully genotyped _k_th locus and *n*_k_ is the number of successfully genotyped loci. Observed adult multilocus heterozygosity (*H*_j_) was also estimated by *H*_j_ = Σ*h*_jk_/*n*_k_. Samples that failed to genotype at four or more of the seven loci (*n* = 1) were excluded from analyses (samples genotyped at five loci *n* = 17).

### Statistical analysis

For each provenance, we used Gaussian general linear models (GLMs) in a maximum likelihood, multi-model inference framework in R v.2.12.1 (R Project for Statistical Computing, http://www.r-project.org; Burnham & Andersen 2002) to test for hypothesised relationships between family-level genetic predictors (*t*_m_, *t*_*m*_ *− t*_*s*_ and *r*_p_) and growth. Additionally, we bootstrapped the regression slopes 10 000 times to assess the validity of these relationships. As mother tree isolation should impact mating systems rather than offspring fitness directly, we did not consider isolation as a statistical factor. To account for potentially confounding environmental variation across populations (Colautti *et al.* 2009), population latitude, mean annual rainfall and mean annual temperature data were integrated into the GLMs. We conducted a principal component analysis to summarise environmental differences between populations, as considerable correlations were present among these environmental variables (see Appendix S2 for intervariable correlations and principal component analysis information). The first principal component explained 52.3% of variation among variables and this component was implemented as a continuous predictor variable in the GLMs. We relied on Akaike’s Information Criterion, corrected for small sample sizes (AIC_*c*_), for model selection (Burnham & Andersen 2002).

Heterozygosity-fitness correlations (HFCs) were investigated for each provenance after Szulkin *et al.* (2010), by initially regressing mean family observed heterozygosity (*Ĥ*_j_) with growth (*ω*_j_ = adjusted*â*_ib_) and estimating the slope (*β*_ωj,*Ĥ*j_) and variance explained (*r*^2^_*ω*j,*Ĥ*j_) by this relationship. As a correlation between heterozygosity and fitness does not indicate how much variation is explained by inbreeding, the inbreeding load (*β*_*ω*j,*f*_) and variance in fitness explained by inbreeding (*r*^2^_*ω*j*,f*_) were also estimated after Szulkin *et al.* (2010). HFCs rely on correlations between observed heterozygosity at genotyped markers and heterozygosity at functional loci (i.e. correlation due to identity disequilibrium) and therefore the interlocus heterozygosity correlation for each provenance (*g*_2_) was estimated in RMES (David *et al.* 2007), where a significant correlation indicates the presence of identity disequilibrium. HFCs also rely on variation in inbreeding, thus the inbreeding estimate, *f*, was derived from the selfing rate, *s*, where *s* = 1 − *t*_m_, and then *f* = *s*/(2 − *s*) (David *et al.* 2007).

## Results

### Variation in mating patterns and genetic diversity

Mating system analysis showed that *S. macrophylla* across Central America was primarily outcrossed and that families from dry provenances were almost entirely outcrossed (Table 1). Mesic provenances exhibited greater correlated paternity than dry provenances. Biparental inbreeding did not differ between provenances.

**Table 1:** Fitness, genetic diversity, and mating system summary data for *Swietenia macrophylla* samples across Central America from contrasting provenances and landscape contexts (*n*_family_, total number of families (i.e. mother trees) per group; *n*_progeny_, total number of progeny across families per group; growth, mean block adjusted growth; *H*_j_, mean observed multilocus heterozygosity of adults; *Ĥ*_j_, mean observed multilocus heterozygosity of progeny; *t*_m_, multilocus outcrossing rate; *t*_m_ − *t*_s_, biparental inbreeding estimate; *r*_p_, multilocus correlated paternity; standard deviations in parentheses; 95% confidence interval homogeneous subgroups indicated by ‘^a^’, ‘^b^’and ‘^c^’)

Group	*n*_family_, *n*_progeny_	Growth (m^3^)	*H*_j_	*Ĥ*_j_	*t*_m_	*t*_m_ − *t*_s_	*r*_p_
*Central America*	71 611	0.056 (0.012)	0.585 (0.24)	0.590 (0.104)	0.968 (0.010)	0.173 (0.020)	0.208 (0.026)
*Provenance*							
Mesic	36 294	0.053 (0.013)^a^	0.520 (0.224)^a^	0.593 (0.139)^a^	0.938 (0.018)^a^	0.198 (0.031)^a^	0.310 (0.045)^a^
Dry	35 317	0.059 (0.010)^a^	0.705 (0.162)^b^	0.632 (0.056)^a^	0.992 (0.033)^b^	0.171 (0.038)^a^	0.170 (0.030)^b^
*Landscape context*							
Forest	47 407	0.060 (0.010)^a^	0.588 (0.224)^a^	0.607 (0.096)^a^	0.991 (0.020)^a^	0.129 (0.026)^a^	0.163 (0.024)^a^
Isolated	24 204	0.048 (0.012)^b^	0.579 (0.255)^a^	0.556 (0.112)^a^	0.925 (0.026)^b^	0.250 (0.032)^b^	0.341 (0.061)^b^
*Provenance and landscape context*							
Mesic forest	24 194	0.059 (0.011)^a^	0.560 (0.214)^a b^	0.604 (0.125)^a^	0.987 (0.042)^a^	0.148 (0.052)^a^	0.254 (0.052)^a^
Mesic isolated	12 100	0.043 (0.012)^b^	0.440 (0.232)^b^	0.503 (0.115)^a^	0.845 (0.041)^b^	0.314 (0.027)^b^	0.445 (0.086)^b^
Dry forest	23 213	0.062 (0.010)^a^	0.687 (0.160)^a^	0.611 (0.055)^a^	0.992 (0.054)^a^	0.149 (0.055)^a^	0.153 (0.026)^c^
Dry isolated	12 104	0.054 (0.010)^a b^	0.740 (0.167)^a^	0.610 (0.082)^a^	0.992 (0.103)^a^	0.208 (0.086)^a^	0.278 (0.084)^a^

Across all Central American populations, isolated trees had markedly disrupted mating patterns with reduced outcrossing rates, increased biparental inbreeding and increased correlated paternity, compared with progeny sampled from a forest context (Table 1). However, the effect was not uniform among provenances. Isolated mesic provenances experienced the most severe disruption to mating patterns, with reduced outcrossing, increased biparental inbreeding and increased correlated paternity. Although, for dry provenances, levels of outcrossing remained unchanged, biparental inbreeding and correlated paternity were elevated in isolated provenances compared with progeny from a forest context. Heterozygosity of adults and offspring was greater in dry provenances than mesic provenances. Heterozygosity of mesic provenance progeny and adults declined with isolation, but both progeny and adults of dry provenances displayed limited change in heterozygosity with isolation.

We detected significant null alleles at two microsatellite loci in both provenances (SM46: 33 and 24%, SM51: 10 and 14%, in mesic and dry provenances respectively), but original and null allele adjusted F_IS_ estimates were similar, thus the empirical data were used for subsequent analyses. Considerable increases in null alleles (e.g. 15% at all loci) were required to give comparable mating patterns and genetic diversity estimates in both provenances (see Appendix S1 for details of null allele results).

### Variation in fitness traits

A total of 611 progeny from 71 families sampled from 16 populations were observed over the five-year common garden experiment. Mesic provenance progeny were significantly smaller than dry provenance progeny (Table 1). Progeny from trees in isolated contexts were significantly smaller than forest context trees and this difference was more pronounced in mesic provenance progeny than dry provenance progeny.

### Correlations between mating system parameters and fitness

Analysed across all Central American populations, increasing outcrossing rate had a strong positive effect on progeny growth, even when controlling for variation in environmental differences among populations (Table 2). Overall, increasing correlated paternity had a negative effect on growth and its effect was equivalent to that of outcrossing rate. Neither biparental inbreeding nor environmental differences showed a relationship with growth when considered in single parameter models.

**Table 2:** General linear models of relationships among genetic and environmental predictors and response variable ‘growth’, a fitness proxy of *Swietenia macrophylla*. Analyses conducted for both isolated and forest landscape context samples grouped to include all samples, samples from only mesic and dry provenances (% DE, percent deviance explained by model *i*; *w*AIC, AIC weights shows the relative likelihood of model *i*; ΔAIC_*c*_, difference between model AIC and minimum AIC in the set of models; AIC_*c*_, Akaike’s Information Criterion corrected for small samples sizes; *k*, number of parameters in the given model; *ß*, unstandardised regression slopes and standard errors for each predictor variable in models with ΔAIC_*c*_ < 4; *t*_m_, outcrossing rate; *t*_m_ − *t*_s_, biparental inbreeding; *r*_p_, correlated paternity; PC_ENV_, first component of principal component analysis of environmental variables; 1, null model)

Model	% DE	*w*AIC	ΔAIC_*c*_	AIC_*c*_	*k*	*ß* (m^3^)
*All families*						
growth ∼ *t*_m_	18.00	0.43	0.00	− 436.70	3	0.052 (0.013)
growth ∼ *r*_p_	16.92	0.27	0.93	− 435.77	3	− 0.024 (0.007)
growth ∼ *t*_m_ + PC_ENV_	23.57	0.18	1.75	− 434.95	6	*t*_m_ = 0.042 (0.014);PC_ENV_ = 0.003 (0.001)
growth ∼ *t*_m_ − *t*_s_	12.93	0.05	4.26	− 432.44	3	
growth ∼ *r*_p_ + PC_ENV_	20.32	0.04	4.71	− 431.98	6	
growth ∼ *t*_m_ − *t*_s_ + PC_ENV_	18.31	0.02	6.48	− 430.22	6	
growth ∼ PC_ENV_	13.39	0.01	8.31	− 428.39	5	
growth ∼ 1	0.00	0.00	11.97	− 424.72	2	
*Mesic provenance*						
growth ∼ *t*_m_	22.91	0.46	0.00	− 215.11	3	0.048 (0.015)
growth ∼ *r*_p_	18.88	0.18	1.83	− 213.27	3	− 0.029 (0.010)
growth ∼ *t*_m_ + PC_ENV_	33.46	0.14	2.39	− 212.72	6	*t*_m_ = 0.035 (0.015);PC_ENV_ = 0.014 (0.006)
growth ∼ *t*_m_ − *t*_s_	15.57	0.09	3.27	− 211.83	3	− 0.034 (0.014)
growth ∼ PC_ENV_	22.99	0.04	4.92	− 210.19	5	
growth ∼ *r*_p_ + PC_ENV_	28.51	0.04	4.98	− 210.13	6	
growth ∼ *t*_m_ − *t*_s_ + PC_ENV_	27.72	0.03	5.37	− 209.74	6	
growth ∼ 1	0.00	0.01	7.11	− 208.00	2	
*Dry provenance*						
growth ∼ *r*_p_	9.92	0.50	0.00	− 223.17	3	− 0.016 (0.008)
growth ∼ 1	0.00	0.25	1.41	− 221.76	2	
growth ∼ PC_ENV_	5.89	0.12	2.90	− 220.27	3	
growth ∼ *r*_p_ + PC_ENV_	9.92	0.09	3.51	− 219.66	3	
growth ∼ *t*_m_ − *t*_s_	2.13	0.02	6.46	− 216.71	5	
growth ∼ *t*_m_	0.42	0.01	7.63	− 215.54	6	
growth ∼ *t*_m_ − *t*_s_ + PC_ENV_	6.07	0.01	9.10	− 214.07	6	
growth ∼ *t*_m_ + PC_ENV_	6.04	0.01	9.11	− 214.06	6	

**Figure 3:**
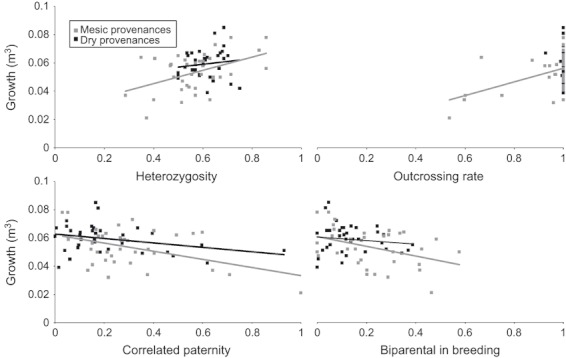
Scatterplots showing relationships between family-level genetic parameters and growth for both provenances. Growth is shown on the *y*-axis and genetic parameter values are shown on the *x-*axis. Mesic provenance family data are indicated by grey-filled squares, dry provenance family data indicated by black-filled squares. Linear trend lines between genetic parameters and growth shown for relationships where ΔAIC_*c*_ < 4 (ΔAIC_*c*_ values presented in Table 2), with grey lines for mesic provenances and black lines for dry provenances.

For mesic provenances, outcrossing rate and correlated paternity explained most variation in progeny growth, even when controlling for environmental differences among populations (Table 2; Fig. 3). Biparental inbreeding was also negatively correlated with growth, but had lower influence than outcrossing rate and correlated paternity. For dry provenances, only the model that included variation in correlated paternity fitted the growth data better than the null model. Outcrossing rate, biparental inbreeding and environmental differences showed no relationship with growth in dry provenances.

As family-level estimates of mating system parameters have higher levels of variance than mating system parameters estimated for groups of families, we ran correlations between population mating system estimates and fitness for each provenance (see Appendix S3 for full details). Additionally, we bootstrapped the regression slopes of the family-level analyses. These analyses all supported our original family-level analyses. Each correlation between population-level mating system parameters and growth was in the same direction as our family-level analyses (Appendix S3). Additionally, the 2.5 and 97.5 percentiles of the bootstrapped slope distributions also confirmed the family-level trends (Appendix S3).

### Correlations between heterozygosity and fitness

Progeny from dry provenances exhibited higher heterozygosity than those from mesic provenances. However, progeny from mesic provenances had greater variation in heterozygosity and displayed greater inbreeding than dry provenances (Table 1, 3). Heterozygosity was positively correlated with growth in both provenances, but this relationship was much stronger in mesic provenances. Interlocus correlation of heterozygosity (i.e. identity disequilibrium), as measured by g_2_, was significant for mesic provenances, but not dry. Consequently, the relationship between inbreeding and fitness (using progeny growth as the quantified variable, *r*^2^_*ωj,f*_) and inbreeding load (*β*_*ωj,f*_) was only estimated for mesic provenances, where inbreeding explained 31.7% of variation in fitness and had an inbreeding load of− 0.042 m^3^. This inbreeding load translates to an average change in fitness after one generation of selfing (i.e*. f* = 1/2) of − 0.042 m^3^ × 1/2 = − 0.021 m^3^.

**Table 3:** HFC comparisons following Szulkin *et al.* (2010) for both mesic and dry provenances of *Swietenia macrophylla* (*Ĥ*_j_ and *σ*^2^(*Ĥ*_j_), heterozygosity mean and variance, respectively; *f*, inbreeding estimate derived from the MLTR selfing rate where *f* = *s*/(2 − *s*); *g*_*2*_, interlocus heterozygosity correlation inferred from RMES (David *et al.* 2007); *r*^2^_*ω*j,*Ĥ*j_, the variation in fitness explained by heterozygosity; *β*_*ω*j,*Ĥ*j_, regression slope of fitness-heterozygosity regression; *r*^2^_*ωj,f*_, variation in fitness explained by inbreeding; *β*_*ωj,f*_, regression slope of fitness-inbreeding, the inbreeding load; variance parameters in parentheses; *g*_*2*_ values followed by ‘^*^’ or ‘^NS^’ indicate significant and non-significant interlocus heterozygosity correlation respectively)

Provenance	*Ĥ*_j_	*σ*^2^(*Ĥ*_j_)	*f*	*β*_*ωj,Ĥ*j_	*r*^2^_*ω*j,*Ĥ*j_	*g*_*2*_	*r*^2^_*ωj,f*_	*β*_*ωj,f*_
Mesic	0.593	0.139	0.032	0.047	0.209	0.033^*^	0.317	− 0.042
Dry	0.632	0.056	0.004	0.020	0.016	− 0.012^NS^		

## Discussion

We demonstrate that across 16 Central American populations of big-leaf mahogany, *S. macrophylla*, a globally threatened species of paramount ecological and economic importance, variation in mating system and progeny growth (used as an indicator of fitness) was significantly impacted by the level of habitat disturbance. Interestingly, populations from mesic regions exhibited greater impact than those from dry regions, indicating the importance of regional or cross-population comparisons when assessing the fitness and mating impacts of habitat disturbance, a factor rarely considered, but often speculated on (Lowe *et al.* 2005; Aguilar *et al.* 2008; Eckert *et al.* 2010; Szulkin *et al.* 2010). Additionally, by tracking growth of progeny over 5 years and by examining individual family (i.e. groups of offspring from a known mother tree) rather than the mean of groups of families, we demonstrate that progeny growth was reduced not only by increased selfing (i.e. inbreeding depression component due to the inbreeding load; Szulkin *et al.* 2010) but also by increased correlated paternity (i.e. via pollen diversity effects; Yasui 1998) – a factor seldom integrated into these types of studies.

### Context-dependent effects of disturbance on mating patterns and genetic diversity

Our data support the wealth of previous studies that document the negative effects of habitat disturbance by increasing inbreeding (Lowe *et al.* 2005; Eckert *et al.* 2010), reducing genetic diversity (Lowe *et al.* 2005; Aguilar *et al.* 2008) and increasing correlated paternity (Lowe *et al.* 2005). Interestingly, the effect of disturbance on mating patterns in our study was provenance-specific. In particular, dry provenances had similarly high levels of progeny heterozygosity and outcrossing in both forest and isolated tree contexts (≤ 0.1% difference). In contrast, mesic provenances demonstrated reduced outcrossing rate and progeny heterozygosity in isolated compared with forest context trees (14 and 10% reduction respectively). In addition, both provenances experienced increased correlated paternity and biparental inbreeding with isolation, but to a much greater extent in mesic compared with dry provenances (*r*_p_ = 0.19 and 0.13 increase, respectively; *t*_m_ − *t*_s_ = 0.17 and 0.06 increase respectively).

Most studies examining the genetic effects of disturbance in tree species have studied only one or a few populations per species (Ward *et al.* 2005; Eckert *et al.* 2010). As such, the stark difference between provenances (i.e. groups of ecologically similar populations) found here highlights the importance of considering intraspecific variation when making either species-wide conclusions or generalities about the effects of disturbance. Upon consideration of multiple populations within a species, which ideally cross known environmental and/or genetic breaks to account for variation in evolutionary history (e.g. by rainfall provenance in this case), more confident extrapolations to species-wide trends can be made. Intraspecific variation in mating systems has been previously discussed (Barrett 2003), but little attention has been applied to contemporary landscape change as a driver for this change. Environmental and genetic breaks may not necessarily correlate as genetic breaks may, for example, follow latitude and environmental breaks may follow longitude (Colautti *et al.* 2009). Thus, efforts to control for population-specific effects are dependent upon *a priori* knowledge about what kind of break is important. Here, quantitative genetic data were used to inform provenance delineation from Navarro & Hernandez (2004) and Navarro *et al.* (2011). Further population genetic structure data would offer greater insight into population effects and both types of data are important considerations when attempting to overcome environmental and genetic effects on fitness. We analysed relative progeny growth among families within provenances (each provenance was analysed separately and environmental data were included in the statistical models to control for environmental effects); therefore, population differences were unlikely to be confounding genetic trends within each provenance (e.g. local adaptation or poor suitability of dry or wet provenances to the experimental conditions).

There are a number of explanations for why we observe a greater reduction in the effective density of pollen donors in mesic compared with dry provenances. First, wetter conditions are less favourable than dry to *S. macrophylla* survival (Lamb 1966; Holdridge *et al.* 1971; Gillies *et al.* 1999). Second, mesic provenances have experienced greater human disturbance. Gillies *et al.* (1999) presented logging intensity data and, using these data, the average logging intensity was higher for mesic provenances in our study than dry (logging intensity: mesic = 0.42; dry = 0.29), but did not increase isolation of mature trees (mean density: mesic provenances = 1.28 trees ha^−1^; dry provenances = 1.23 trees ha^−1^). These factors will serve to reduce the effective density of mahogany trees in mesic compared with dry habitats and drive greater genetic impacts in the former compared with the latter. Indeed, mesic provenances exhibit lower genetic diversity (adult heterozygosity: mesic = 0.520; dry = 0.705; progeny heterozygosity: mesic = 0.593; dry = 0.632) and increased inbreeding (selfing: mesic = 6.2%; dry = 0.8%; biparental inbreeding: mesic = 0.198; dry = 0.171) compared with dry provenances. Reduced population genetic diversity is also expected to lead to a decline in genetic diversity of pollen clouds, which will be reflected as higher correlated paternity values (correlated paternity: mesic = 0.310; dry = 0.170). Third, it is plausible that mesic provenances have a different pollinator community dominated by less mobile pollinators, resulting in stronger mating pattern shifts with disturbance. If less mobile pollinators were present in mesic provenances, we would expect elevated levels of selfing in these provenances as less mobile pollinators would spend more time in individual canopies, increasing selfing rates (Ottewell *et al.* 2009) and correlated paternity in isolated provenances (an effect we observe). While less mobile pollinators in mesic provenances may be contributing to the observed mating patterns, there is currently little information on the relative pollinator abundance and behaviour in these different provenances. These pollinator effects would synergistically emphasise the effect of small population sizes (as outlined above) rather than counter them. Further studies on pollinator dynamics in these regions is recommended as follow-up work.

Independent of the mechanism, higher correlated paternity and reduced effective pollen donor density are expected to lead to a shift in reproductive assurance strategies and costs of inbreeding, as occasional selfing would be favoured over reproductive failure (as long as selfing was allowed), even if inbreeding depression was significant (Herlihy & Eckert 2002; Barrett 2003; Kalisz *et al.* 2004). However, ephemeral changes to pollen clouds are unlikely to result in strong directional selection for increased mixed mating, although directional or balancing selection for increased mixed mating may indeed occur in chronically fragmented landscapes due to mate limitations (Kennedy & Elle 2008; Winn *et al.* 2011). Levels of selfing observed in isolated mesic provenances in our study (selfing rate = 15%) are approximately twice that observed by Lemes *et al.* (2007) in a study of a logged Brazilian population of *S. macrophylla* (selfing rate = 7%). Lack of selfing in dry provenances from Central America is likely due to higher effective pollen donor density, resulting in preferential outcrossing and reproduction both being assured without requiring an increase in selfing.

### Mating system-fitness and heterozygosity-fitness relationships

We demonstrate that family-level reductions in outcrossing rate and increases in correlated paternity have strong fitness costs (at least at the stage of older saplings, which were used as a surrogate to measure fitness in this study) for isolated mahogany trees and are provenance-dependent. Families from mesic provenances experienced significant selfing and greater variance in heterozygosity and, accordingly, we observed a significant interlocus correlation of heterozygosity (g_2_ = 0.033) and a correlation between inbreeding and fitness (Szulkin *et al.* 2010). In contrast, families from dry provenances were almost completely outcrossed and had low variance in heterozygosity, therefore were not expected to express an interlocus correlation of heterozygosity or a relationship between inbreeding and fitness. Variation in pollen cloud diversity, as measured by correlated paternity, appears to be a major factor reducing fitness in dry provenances. Further work needs to be undertaken to assess the potential for fitness effects to be expressed at life stages other than progeny growth (e.g. pollination and germination). However, the ability for saplings to quickly capture a site is a significant advantage for this pioneer species and is a life stage that selection pressures are expected to be strong.

The few studies where correlated paternity has been examined in conjunction with fitness warrant discussion here, but represent data from single populations and fitness measurements taken over short time-frames. Cascante *et al.* (2002) reported that *Samanea saman* trees occurring in low density tended to have higher correlated paternity than trees in high densities. This difference in mating pattern was associated with poorer progeny vigour (measured 45 days post-germination). Fuchs *et al.* (2003) found that isolated *Pachira quinata* trees had higher levels of correlated paternity than trees in continuous forest. These isolated trees had lower flower-to-fruit set conversion rates, which were hypothesised to be due to limited compatible pollen received by isolated trees. In contrast, Rocha & Aguilar (2001) found that isolated *Enterolobium cyclocarpum* trees had lower correlated paternity than high-density trees, yet progeny from isolated trees generally had lower vigour. The authors suggested that this result might be due to fewer opportunities for selective abortions (i.e. less pollen competition) in pasture trees because less pollen was received. The difference in fitness could equally be explained by early-acting maternal effects, particularly as seed from forest trees were significantly larger than those from pastures (Charlesworth 1988).

Greater pollen diversity should facilitate the acquisition of more ‘good genes’ within a progeny array and is expected to be driven through pollen competition by providing a forum to remove individuals carrying deleterious recessive alleles (Charlesworth 1988; Yasui 1998; Armbruster & Gobeille 2004). The findings of our study fit this hypothesis, where, in dry provenances, greater pollen competition (i.e. lower correlated paternity) potentially mitigates fitness impacts of fragmentation. In contrast, mesic provenances probably experienced far less pollen competition, as evidenced by high correlated paternity, and exhibited a strong relationship between inbreeding and fitness. Without examining pollen tube growth rates, we cannot exclude the possibility that these inbreeding-fitness trends were only the result of greater inbreeding in wet provenances.

## Conclusions and future directions

We link variation in family-level mating system patterns with fitness observed over 5 years for *S. macrophylla* across 16 Central American populations. Our results illustrate the first case of dramatic intraspecific variation in mating system and fitness responses to habitat disturbance, as well as shifts in reproductive assurance and inbreeding costs in different portions of the range of a species. Consequently, we strongly recommend caution when extrapolating limited mating system data to species-wide conclusions.

These results also have important ecological and applied implications (Fig. 1). Mahogany seed sourced from disturbed landscapes throughout Central America is likely to be lower quality than seed sourced from intact forest, leading to poorer outcomes for agroforestry and revegetation projects. This work also highlights the need to protect remnant forest resources and develop appropriate provenance sourcing strategies for restoration plantings (Broadhurst *et al.* 2008; Sgrò*et al.* 2011).

## Supporting information

As a service to our authors and readers, this journal provides supporting information supplied by the authors. Such materials are peer-reviewed and may be re-organized for online delivery, but are not copy-edited or typeset. Technical support issues arising from supporting information (other than missing files) should be addressed to the authors.
