# Supplementary material for: Shifts in reproductive assurance strategies and inbreeding costs associated with habitat fragmentation in Central American mahogany
Source: Ecol Lett. 2012 May;15(5):444–52. doi: 10.1111/j.1461-0248.2012.01752.x (PMC3489046; doi:10.1111/j.1461-0248.2012.01752.x)
Supplement: Supplementary file 1 [file ele0015-0444-SD1.doc]

**Appendix S1.** Results of null allele analyses

Two loci (SM46 and SM51) had significant levels of null alleles detected under HWE expectations for both mesic and dry provenances. The null allele frequency of SM46 was on average 9% higher in mesic than dry provenances (33 to 24 %), and the null allele frequency of SM51 was on average 4% higher in dry than mesic provenances (14 to 10%). Considerable variation in F_IS_ estimates was observed across loci in both provenances, however F_IS_ estimates were similar with and without null allele adjusted datasets, thus the observed data were used for all subsequent analyses (Table S3). By simulating increases in null alleles in dry provenances (i.e. dry provenances were more diverse and more outcrossed - see results and Table 1), drastic increases in null allele frequencies were needed to give comparable genetic parameter estimates in both provenances (Table S4). For example, outcrossing rate (*t*_m_) and correlated paternity (*r*_p_), the key mating system parameters discussed in our study, did not converge even when random null allele increases occurred at 15% in dry provenances at 6 of the 7 loci.

Table S1. F_IS_ estimates of observed and null allele adjusted datasets across loci for both *Swietenia macrophylla* provenances. Where significant null alleles were detected in MICRO-CHECKER, F_IS_ estimates were made on null allele adjusted datasets and reported in parentheses after the observed estimate.

| Locus | Dry provenances | Mesic provenances |
| --- | --- | --- |
| SM01 | -0.03 | 0.12 |
| SM22 | 0.00 | -0.03 |
| SM31 | 0.17 | 0.24 |
| SM32 | -0.19 | 0.17 |
| SM40 | -0.01 | 0.29 |
| SM46 | 0.40 (0.39) | 0.51 (0.49) |
| SM51 | 0.25 (0.17) | 0.20 (0.15) |
| Overall | 0.07 | 0.21 |

Table S2. Observed heterozygosity and mating system data for both provenances as well as simulated values with artificially inflated levels of null alleles. For heterozygosity, the percentage increase in the levels of null alleles in the dry provenance at a given number of loci to get equivalent heterozygosity as mesic provenances is shown. For mating system parameters, the parameter estimates are reported for the given number of dry provenance loci that had their null allele frequencies artificially increased by 15% (*H*_j_, mean observed multilocus heterozygosity of adults; *Ĥ*_j_, mean observed multilocus heterozygosity of progeny; *t*_m_, multilocus outcrossing rate; *t*_m_-*t*_s_, biparental inbreeding estimate; *r*_p_, multilocus correlated paternity; standard deviations in parentheses).

| Provenance or loci group | *H*_j_ | *Ĥ*_j_ | *t*_m_ | *t*_m_-*t*_s_ | *r*_p_ |
| --- | --- | --- | --- | --- | --- |
| *Observed data* |  |  |  |  |  |
| Mesic | 0.520 (0.224) | 0.593 (0.139) | 0.938 (0.018) | 0.198 (0.031) | 0.310 (0.045) |
| Dry | 0.705 (0.162) | 0.632 (0.056) | 0.992 (0.033) | 0.171 (0.038) | 0.170 (0.030) |
| *Simulated data* |  |  |  |  |  |
| 1 locus | Inf. | 23% | Not performed | Not performed | Not performed |
| 2 loci | 65% | 12% | Not performed | Not performed | Not performed |
| 3 loci | 44% | 8% | 0.970-0.987 | 0.186-0.253 | 0.193-0.235 |
| 4 loci | 32% | 6% | 0.951-0.970 | 0.230-0.278 | 0.224-0.247 |
| 5 loci | 26% | 5% | 0.951-0.966 | 0.255-0.273 | 0.232-0.247 |
| 6 loci | 21% | 4% | 0.947 | 0.282 | 0.239 |
| 7 loci | 18% | 3% | Not performed | Not performed | Not performed |
