# Supplementary material for: Shifts in reproductive assurance strategies and inbreeding costs associated with habitat fragmentation in Central American mahogany
Source: Ecol Lett. 2012 May;15(5):444–52. doi: 10.1111/j.1461-0248.2012.01752.x (PMC3489046; doi:10.1111/j.1461-0248.2012.01752.x)
Supplement: Supplementary file 2 [file ele0015-0444-SD2.doc]

**Appendix S2.** Results of principal component analysis and population environmental variable correlations.

Table S3. Principal component analysis of *Swietenia macrophylla* population environmental variables latitude, rainfall and temperature. Table shows significant component eigenvalues and variance explained, correlation coefficient between principal components and variables included in PCA (correlation significance shown by ^NS^, *, **, *** for *p*-values >0.05, <0.05, <0.01 and <0.001, respectively). PC1 was used as a covariate predictor variable for models of generalised linear modelling effects of mating system parameters (e.g. outcrossing rate, correlate paternity) and fitness.

| **Component** | **Eigenvalue** | **Variance explained (%)** | **Rainfall *r*** | **Latitude *r*** | **Temperature *r*** |
| --- | --- | --- | --- | --- | --- |
| PC1 | 1.57 | 52.31 | -0.90*** | -0.85*** | 0.20^NS^ |
| PC2 | 1.05 | 34.89 | -0.10^NS^ | -0.34** | 0.96*** |

Table S4. Correlations between *Swietenia macrophylla* population environmental variables latitude, rainfall and temperature.

| **Correlation** | ***n*** | ***r*** | ***p*-value** |
| --- | --- | --- | --- |
| rainfall ~ latitude | 71 | -0.56 | <0.001 |
| rainfall ~ temperature | 71 | -0.20 | 0.10 |
| latitude ~ temperature | 71 | -0.07 | 0.54 |
