# Supplementary material for: Shifts in reproductive assurance strategies and inbreeding costs associated with habitat fragmentation in Central American mahogany
Source: Ecol Lett. 2012 May;15(5):444–52. doi: 10.1111/j.1461-0248.2012.01752.x (PMC3489046; doi:10.1111/j.1461-0248.2012.01752.x)
Supplement: Supplementary file 3 [file ele0015-0444-SD3.doc]

**Appendix S3.** Family-level GLM variance testing and population-level trends

Since family-level estimates of mating system parameters have higher levels of variance than mating system parameters estimated for groups of families, we ran correlations between population mating system estimates and fitness for each provenance and we bootstrapped the regression slopes of the family-level analyses 10000 times in R v. 2.12.1 (R Project for Statistical Computing, http://www.r-project.org). We bootstrapped the regression slopes of each family mating system parameter determined to be important in predicting growth (based on the original GLM modelling where the important parameters were included in the models that were either the best fitting model or had a ΔAICc <4 but < than the ΔAICc of the null model).

Table S5. Fitness, genetic diversity and mating system summary data for *Swietenia macrophylla* from each population samples across Central America (*n*_family_, total number of families (i.e. mother trees) per group; *n*_progeny_, total number of progeny across families per group; growth, mean block adjusted growth; *t*_m_, multilocus outcrossing rate; *t*_m_ - *t*_s_, biparental inbreeding estimate; *r*_p_, multilocus correlated paternity; standard deviations in parentheses).

| Population | Provenance | *n*_family_, *n*_progeny_ | Growth (m^3^) | *t*_m_ | *t*_m_-*t*_s_ | *r*_p_ |
| --- | --- | --- | --- | --- | --- | --- |
| Cano Negro | Mesic | 2, 20 | 0.037 | 0.90 (0.16) | 0.27 (0.16) | 0.33 (0.12) |
| Coiba | Mesic | 5, 46 | 0.054 | 0.96 (0.14) | 0.17 (0.09) | 0.20 (0.06) |
| Lacentilla | Mesic | 12, 115 | 0.058 | 0.95 (0.06) | 0.13 (0.13) | 0.18 (0.04) |
| Las Cuavas | Mesic | 2, 14 | 0.065 | 1.00 (0.43) | 0.37 (0.17) | 0.24 (0.24) |
| Maramamba | Mesic | 1, 10 | 0.065 | 1.00 (0.26) | 0.47 (0.22) | 0.64 (0.29) |
| Mukuwas | Mesic | 15, 109 | 0.052 | 0.96 (0.03) | 0.24 (0.05) | 0.30 (0.08) |
| San Emilio | Mesic | 3, 30 | 0.052 | 0.90 (0.14) | 0.28 (0.28) | 0.68 (0.22) |
| Abangares | Dry | 1, 10 | 0.050 | 1.00 (0.29) | 0.59 (0.29) | 0.45 (0.22) |
| Bethel | Dry | 4, 37 | 0.066 | 0.98 (0.22) | 0.11 (0.11) | 0.10 (0.06) |
| Bio Itlza | Dry | 1, 10 | 0.072 | 1.00 (0.26) | 0.25 (0.12) | 0.07 (0.45) |
| Chapernal | Dry | 5, 41 | 0.050 | 0.98 (0.12) | 0.34 (0.09) | 0.58 (0.16) |
| Madrazo | Dry | 3, 29 | 0.062 | 1.00 (0.26) | 0.27 (0.08) | 0.22 (0.10) |
| Naranjal | Dry | 4, 36 | 0.064 | 1.00 (0.21) | 0.27 (0.07) | 0.14 (0.07) |
| Nuevo Becal | Dry | 9, 90 | 0.059 | 1.00 (0.06) | 0.08 (0.06) | 0.14 (0.04) |
| San Filipe | Dry | 4, 35 | 0.054 | 1.00 (0.00) | 0.26 (0.08) | 0.04 (0.23) |

Table S6. Bootstrapped slope distributions of family-level regressions showing 2.5 and 97.5 % percentiles.

| Model | Slope | 2.5% | 97.5% |
| --- | --- | --- | --- |
| *All families* |  |  |  |
| growth ~ *t*_m_ | 3.44 | 0.35 | 6.25 |
| growth ~ *r*_p_ | -6.94 | -11.34 | -2.14 |
| growth ~ *t*_m_-*t*_s_ | -4.06 | -6.00 | -1.71 |
| *Mesic provenance* |  |  |  |
| growth ~ *t*_m_ | 4.73 | 0.28 | 8.30 |
| growth ~ *r*_p_ | -6.54 | -11.36 | -0.29 |
| *Dry provenance* |  |  |  |
| growth ~ *r*_p_ | -6.33 | -14.95 | -0.08 |

Table S7. Correlations of population-level mating system estimates and growth (Pearson’s correlation coefficient shown with significance indicated by ^NS^, *, ** for *p*-values >0.05, <0.05 and <0.01, respectively).

|  | All | Dry | Mesic |
| --- | --- | --- | --- |
| growth ~ *t*_m_ | +0.66** |  | +0.86* |
| growth ~ *t*_m_-*t*_s_ |  |  | +0.42^NS^ |
| growth ~ *r*_p_ | -0.36^NS^ | -0.71* | +0.08^NS^ |
